# IgG4-Related Disease coexisting with Behçet’s Disease

**DOI:** 10.31138/mjr.30.4.228

**Published:** 2019-12-31

**Authors:** Mohammed B. Alanazi, Yahya O. Asiri, Ibrahim A. Al-Homood

**Affiliations:** Medical Specialties Department, Rheumatology Section, King Fahad Medical City, Riyadh, Saudi Arabia

**Keywords:** IgG4, Behçet’s, disease, retroperitoneal mass

## Abstract

We describe a case of Immunoglobulin G4-related disease (IgG4-RD) coexisting with Behçet’s disease. A 49-year-old man, with a diagnosis of Behçet’s Disease for 15 years who was found to have an acute kidney injury. His investigations revealed an elevated IgG4 level and the abdominal computerized tomography showed a retroperitoneal mass, which was diagnosed to be IgG4-RD based on histology. The patient showed symptomatic and radiological improvement after starting high dose steroid for 1 month followed by a maintenance dose. Our case report suggested that the two diseases arose separately.

## INTRODUCTION

Immunoglobulin G4-related disease (IgG4-RD) has been recently reported and increasingly recognized in the medical literature as a collection of immune mediated disorders that share the same pathological, serological, and clinical features.^1,2^ It has been associated with diseases and conditions that characterized with prolonged inflammatory process and variable degrees of fibrosis and Lymphoplasmacytic infiltrates, such as Autoimmune Pancreatitis, Sclerosing Cholangitis, Mikulicz Disease, Riedel’s thyroiditis (IgG4-related thyroid disease), IgG4-related kidney disease.^2^ The aim of this report is to illustrate a possible coexistence of these two conditions, drawing attention to the necessity for biopsy of suspecting lesions in patients with Behçet’s disease.

## CASE PRESENTATION

A 49-year-old man, diagnosed with Behçet’s disease 15 years ago, according to the international criteria for Behçet’s disease (ICBD), with recurrent painful oral ulcers, genital ulcer, left lower limb deep venous thrombosis and eye involvement, admitted with progressive dull epigastric pain for two months, with no aggravating or relieving factors. He denied any other complaints. Clinical examination reveals high blood pressure, 188/115 mmHg, with no history of previous hypertension and epigastric tenderness.

Laboratory tests reveal acute raised plasma creatinine (CR) 289 umol/L (baseline is 99 umol/L, reference range 60–100 umol/l), plasma serum Urea 14.2 mmol/l (2.5 to 7.1 mmol/L reference), and C-Reactive Protein (CRP) 12.1 mg/l (reference range < 3.0 mg/L).

Immunoglobulins (Ig) levels were: IgG 17.5 g/L (reference range 7–16 g/L), IgA 3.2 g/L (reference range 0.7–4 g/L), IgM 0.8 g/L (reference range 0.4–2.30 g/L). Serum IgG subclasses levels were: IgG 1 = 11.8 g/L, IgG 2 = 3.7 g/L. IgG 3 = 0.9 g/L. IgG 4 = 0.9 g/L.

Complete blood counts (CBC) and coagulation profile were normal.

Ultrasound of kidneys and bladder reveal bilateral hydro-nephrosis. Computerised tomography (CT) of the abdomen and pelvis without contrast (*Figure 1A*) showed a large retroperitoneal mass at lower lumbar levels, encasing retroperitoneal vessels and ureters measured 7.168 × 3.25 cm with bilateral moderate hydronephrosis. Double J stent was inserted.

**Figure 1A.**
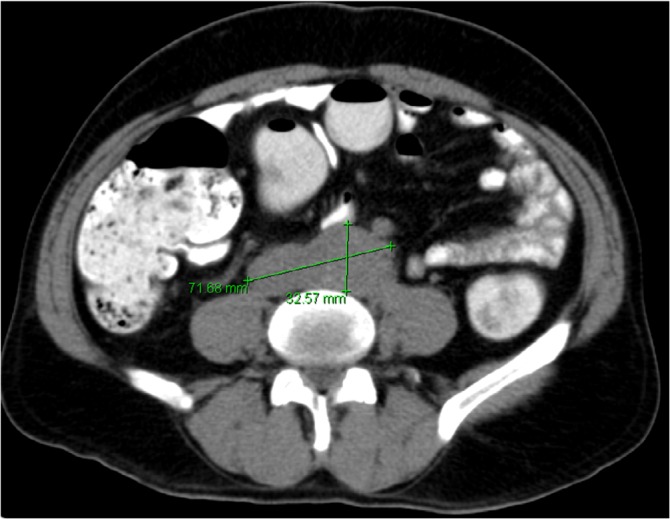
Initial Computerised tomography (CT) of the abdomen and pelvis without contrast showed a large retroperitoneal mass at lower lumbar levels encasing retroperitoneal vessels and ureters measured 7.168 X 3.25 cm with bilateral moderate hydronephrosis.

**Figure 2.**
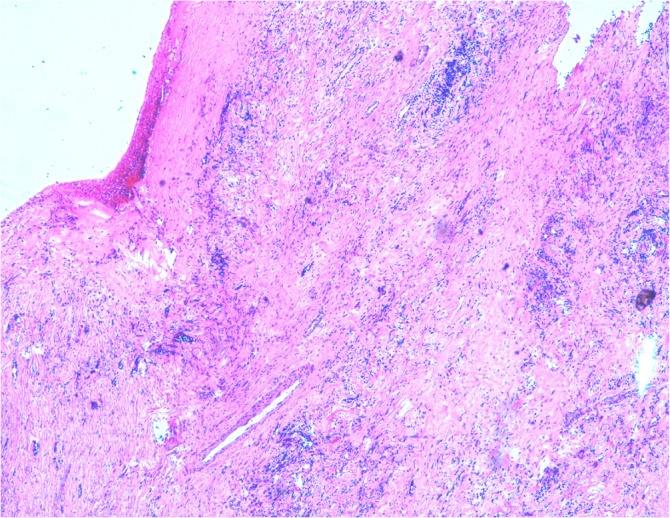
Chronic inflammation with lymphoplasmocytic infiltration. (H&E stain 100×)

Retroperitoneal mass biopsy revealed fibro-adipose tissue infiltrated by moderate amount of inflammatory cells, predominantly of plasma cells and lymphocytes with scattered eosinophils present in the dense collagenous stroma. Formation of lymphoid follicles were not observed, features of retroperitoneal fibrosis, which support Ig-G4 related disease (*Figure 2*).

He was managed initially with amlodipine 10 mg orally daily and high dose of prednisolone 60 mg orally daily for 1 month, followed by slow tapering doses. Five months later, the patient’s symptoms improved significantly and his abdominal CT was repeated, which showed a significant reduction of the retroperitoneal mass (*Figure 1B*). His Behçet’s disease is currently stable on Azathioprine and Colchicine. Patient’s consent was obtained.

**Figure 1B.**
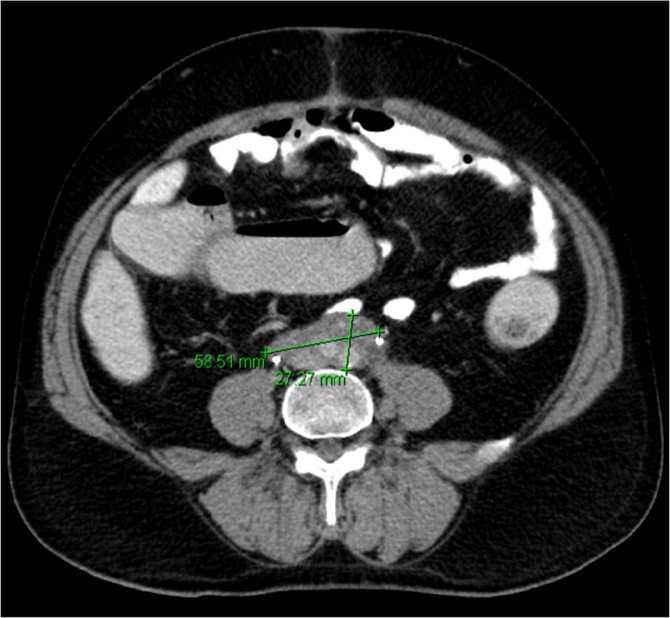
A follow-up CT abdomen that revealed improvement and reduction of retroperitoneal mass.

## DISCUSSION

We reported a case of IgG4-RD in a patient with Behçet’s Disease. Behçet’s disease has heterogeneous manifestations and characterized by relapsing remitting episodes. IgG4-RD has been associated with diseases and conditions with prolonged inflammatory process and variable degrees of fibrosis and lymphoplasmacytic infiltrates.^3–4^ However, it is rarely reported to be coexisted with Behçet’s disease. Shaib et al. reported a patient with Behçet’s disease and laryngeal mass that was finally diagnosed with IgG4-RD with rapid resolution of symptoms with steroid therapy.^3^

Until today, possibly due to lack of studies on the condition, there is no clear universally-accepted hypothesis on the pathogenesis of IgG4-RD and whether the increase in IgG4 is due to primary immune system abnormality or secondary cause as a result of persistent antigenic stimulation.^3^

However, the most likely accepted theory with characteristic pathogenesis is the increased level of T-helper 2 (Th2) lymphocyte, T regulatory cells, and Interleukin-10(IL-10), transforming growth factor-beta (TGF-B), which leads to B-cell increase differentiation of IgG4, among other immunoglobulins types like IgM, IgE, IgG to IgG 4.^4^

TGF-4 leads to increased activity of fibroblasts and myofibroblasts that leads to increased deposition of fibrous tissue in different parts of the body, exerting tumour-like mass effects, resulting in different presentations.^5^

While the condition occurs more in middle-aged men, the epidemiology of the condition remains largely undefined, due to limited cases, variable presentation and variable association with different autoimmune conditions, resulting in difficult diagnosis of the condition.^7^

Because of the wide range of clinical manifestations of IgG4-RD, the diagnosis is often challenging and needs a confirmation by histopathological findings of the affected organ. Glucocorticoids are the main treatment for IgG4-RD. Immunosuppressive agents as steroid-sparing agents are used in patients with refractory disease. Glucocorticoid is prescribed to our patient, to reduce the burden of mass effect as cause of post-renal obstruction with an excellent response.^8^ Azathioprine and Rituximab could be used in cases of relapse or poor response to steroid.^9^

To our knowledge, our case is the first reported case of IgG4-RD retroperitoneal mass in a patient with Behçet’s disease.

## CONCLUSION

Patients with Behçet’s disease with tumefactive lesions should be biopsied, and IgG4-RD should be considered. Early recognition and starting of corticosteroid therapy will diminish unnecessary invasive and risky therapeutic interventions.
